# Melanocytic Matricoma: A Potential Mimic of Malignant Melanoma

**DOI:** 10.7759/cureus.36907

**Published:** 2023-03-30

**Authors:** John Yablonski, Heather Foley, Harleen K Sidhu, Jagmohan S Sidhu

**Affiliations:** 1 Pathology, Upstate University Hospital, Syracuse, USA; 2 Plastic and Reconstructive Surgery/Hand Surgery, United Health Services, Binghamton, USA; 3 Pathology and Laboratory Medicine, United Health Services, Binghamton, USA

**Keywords:** melanoma, dermal tumor, immunohistochemistry staining, histology, melanocytic matricoma

## Abstract

Melanocytic matricoma is a rare dermal tumor that typically presents on the sun-damaged skin of older patients. While there is controversy in the literature regarding the proper characterization of this tumor, there are certain histological and immunohistochemical features that have been described. This report presents a case of melanocytic matricoma with several unusual features that were initially feared to be malignant melanoma. Careful histologic and immunohistochemical analysis was required to rule out malignant melanoma and make the correct diagnosis. Given the rarity of melanocytic matricoma and the potential for it to mimic malignant melanoma, it is important for pathologists to keep melanocytic matricoma on the differential and be aware of the clinical, histological, and immunohistochemical features of this tumor.

## Introduction

Melanocytic matricoma is an extremely rare, biphasic tumor that presents most commonly as a pigmented papule on the sun-damaged skin of older patients [1]. It was first described by Carlson et al. in 1999 as a biphasic tumor harboring both melanocytic and epithelial components [2]. Histology typically shows a nodule within the dermis that contains beta-catenin-positive/LEF1-positive basaloid (blue) matrical cells, shadow (ghost) cells, and dendritic melanocytes that are deeply pigmented [1,3]. Melanocytic matricomas have histopathological and molecular features closely related to pilomatricomas; in fact, there have been attempts to redefine melanocytic matricoma as a special variant of pilomatricoma [1,4]. This report presents a case of melanocytic matricoma and discusses its potential to mimic malignant melanoma.

## Case presentation

The patient was a 64-year-old male with Fitzpatrick skin type III and a dermatologic past medical history most significant for nodular basal cell carcinoma on the left zygoma that was treated with complete excision and an actinic keratosis that was treated with 5% fluorouracil. The patient reported medium lifetime sun exposure and stated that he burns easily; however, the patient denied a history of blistering sunburns. The patient denied a history of artificial tanning bed use and stated that he practiced sun safety, including wearing sunscreen, hats, and sunglasses while outdoors. On routine skin examination, there was a newly noted lesion on the left forehead that had been present for the last few weeks. Dermatoscope examination revealed a pigmented blue/black lesion measuring approximately 1 mm. Initial differential diagnoses included pigmented basal cell carcinoma, early evolving melanoma, and benign blue nevus. Four weeks after identification, the lesion was excised using a 2 mm punch blade. The patient was discharged in good condition and with instructions for follow-up care.

**Figure 1:**
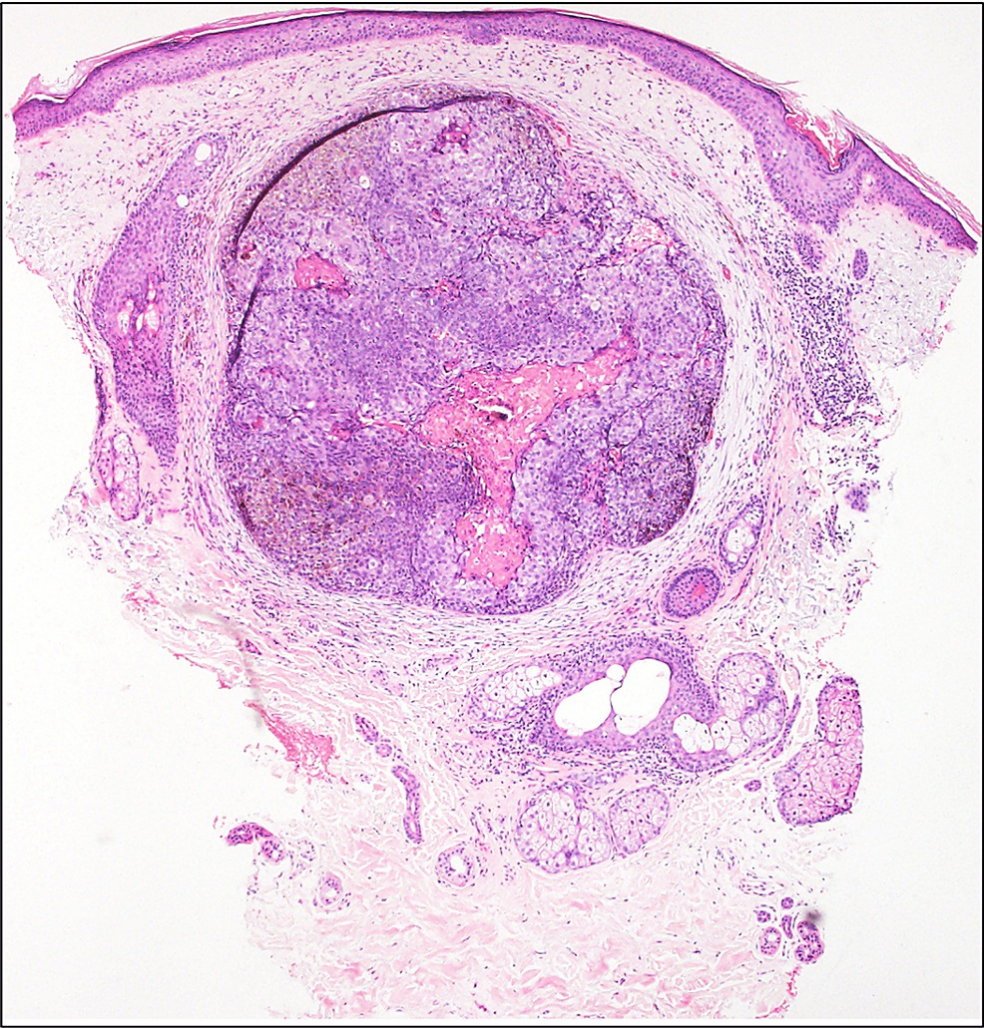
H&E: x40; small (2 mm) circumscribed tumor in the dermis with peripheral patches of pigmented cells

**Figure 2:**
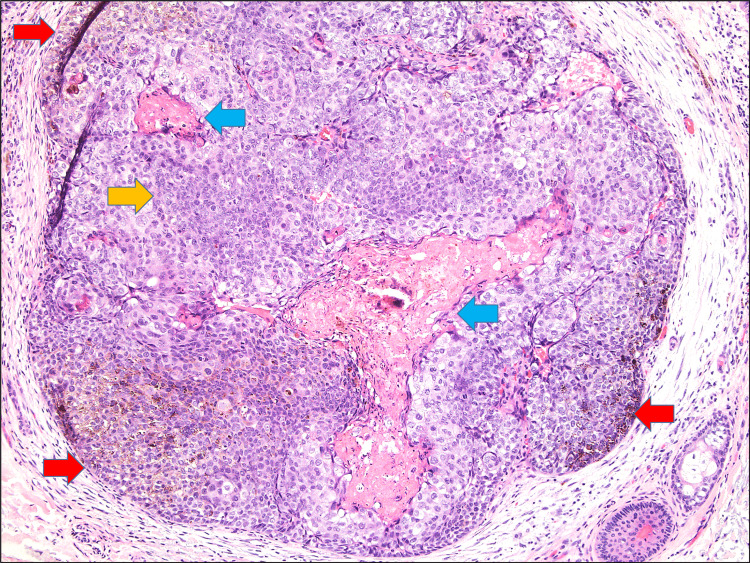
H&E: x100; patches of pigmented cells (red arrows), blue basaloid cells (yellow arrow), and shadow cells (blue arrows)

**Figure 3:**
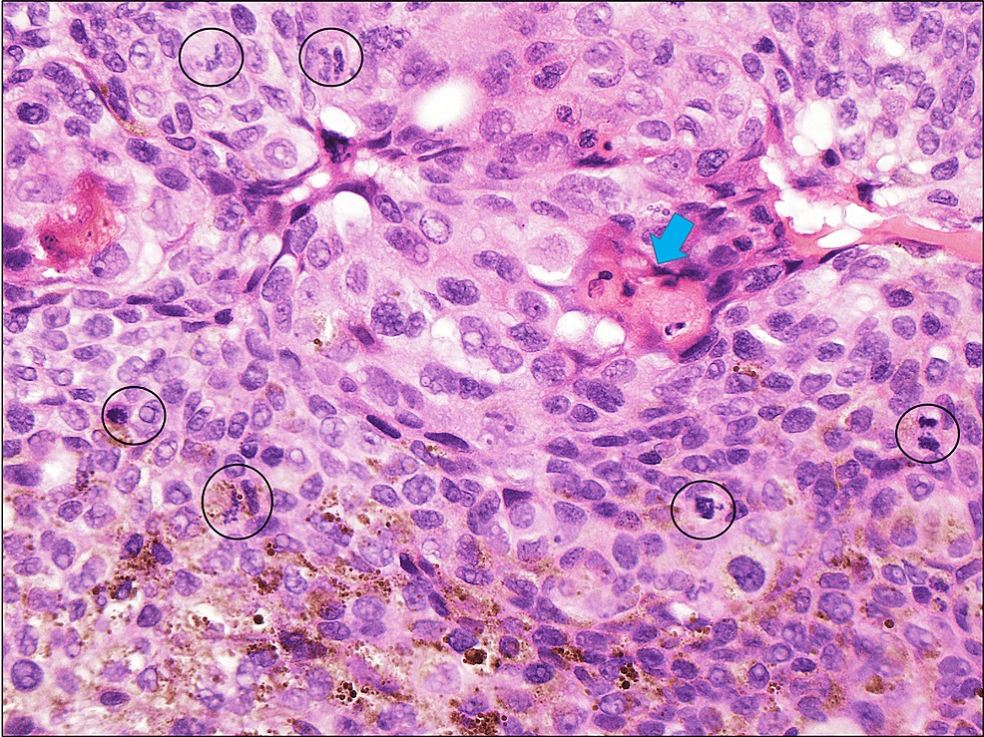
H&E: x400; mitoses close to and away from pigmented cells (black rings) and a few shadow cells (blue arrow)

**Figure 4:**
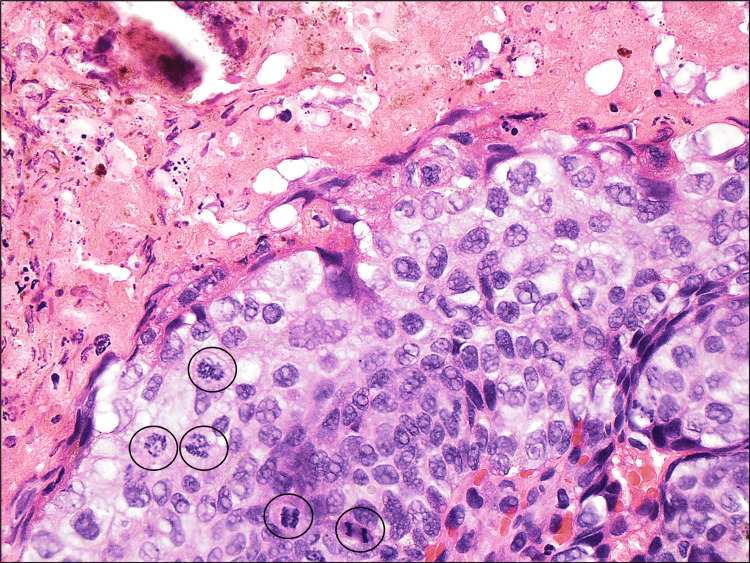
H&E: x400; mitoses in the non-pigmented cells (black rings) and several shadow cells (pink areas)

**Figure 5:**
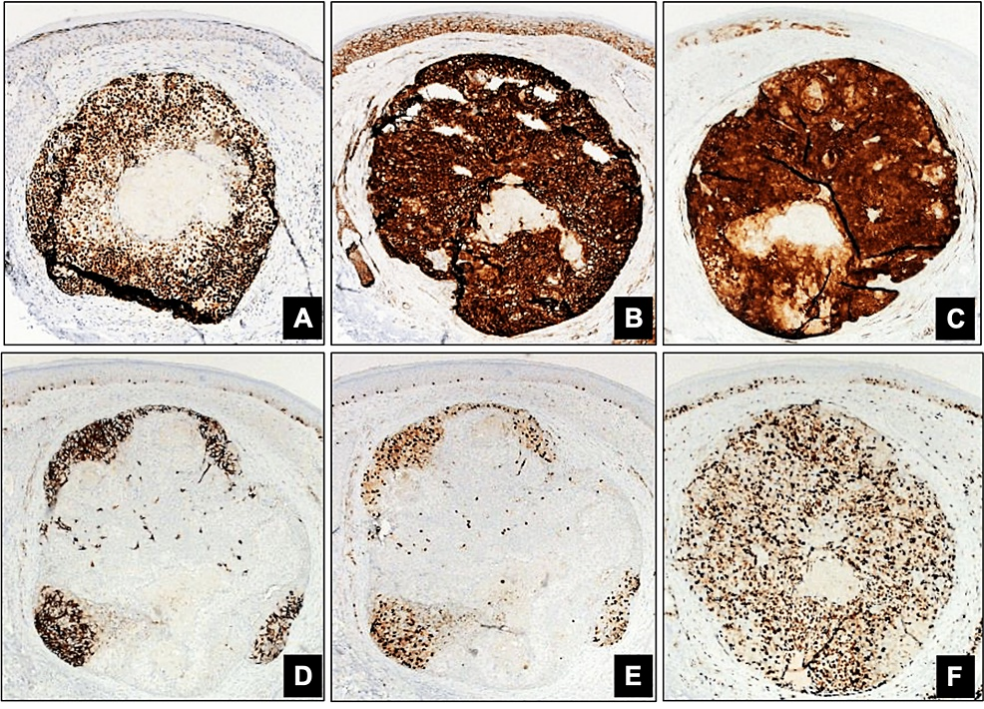
Immunohistochemical stains: x40: A: LEF-1; B: Beta-Catenin; C: p16; D: Melan-A; E: SOX10; F: Ki67

The sample was received in formalin and presented grossly as a tan, wrinkled, circular portion of skin measuring about 2 mm in diameter and excised to a depth of 3 mm. The sample displayed an eccentric slightly smaller than 2 mm purple-brown, ovoid lesion that was <1 mm of the closest surgical margin. Microscopically, the epidermis was unremarkable. Within the dermis, there was a small round neoplasm (slightly smaller than 2 mm) with a central small area of shadow cells surrounded by basaloid epithelial proliferation with peripheral patches of melanin pigment deposition in atypical epithelioid cells (Figure 1). Moderate pleomorphism and hyperchromatism with increased mitotic activity (up to 6 mitoses/high power field average 3-4 mitoses/high power field) were observed predominantly in the non-pigmented epithelial cells and there was a clear-cut abrupt transition of basaloid (blue) cells into shadow cells (Figures 2-4). Immunohistochemical analysis showed that basaloid (blue) epithelial cells were positive for EMA, LEF1 (Figure 5A), beta-catenin (also positive in pigmented cells; Figure 5B), and p16 (also positive in pigmented cells; Figure 5C). Melan-A (Figure 5D), Sox10 (Figure 5E), HMB-45, p16 (Figure 5C), and beta-catenin (Figure 5B), were positive in the areas with melanin pigment. PRAME, adipophilin, and androgen receptors were all negative in the neoplasm. The Ki-67 labeling index was approximately 80% in the entire tumor (Figure 5F). A diagnosis of melanocytic matricoma was made. The lesion site was conservatively re-excised because of the close proximity of the lesion to the margin of the biopsy specimen and no further lesion was seen in the re-excised specimen.

## Discussion

The literature on melanocytic matricomas is scarce. In the existing literature, there has been some controversy regarding the proper characterization of this tumor. Some have suggested that melanocytic matricomas are either a subtype of pilomatricoma or exist on a spectrum, including pilomatricomas [1,5]. There are similar histopathologic and molecular features between melanocytic matricomas and pilomatricomas; however, there have been inconsistent differences between these entities reported in the literature. For example, while melanocytic matricomas are typically seen as a single, well-circumscribed nodule, there have been cases reported that show cystic degeneration, which is usually seen in pilomatricomas [1,5,6]. In addition, there have been cases reported where the melanocytic matricoma was continuous with the epidermis, which is another feature seen in pilomatricomas [1,5,6]. This underscores the importance of further identification and characterization of melanocytic matricomas.

Regardless of the controversy in the characterization of melanocytic matricomas, there have been certain clinical and histological features that have been properly elucidated. The lesion tends to appear on the sun-damaged skin of older patients and presents as a pigmented papule [1,4,6]. A review of the literature showed that lesions are typically 0.20 to 1.70 cm [1]. The case presented in this paper (slightly smaller than 2 mm) is thus an example of an unusually small melanocytic matricoma.

Histologically, melanocytic matricomas present as well-circumscribed, unencapsulated lesions that are usually (but not always) discontinuous with the overlying epidermis [1]. The tumor contains basaloid cells (round nuclei, stippled chromatin, prominent nucleoli) that transition into shadow (ghost) cells (anucleate, eosinophilic, keratinized cytoplasm) in a manner that can be either abrupt or gradual [1]. The tumor also contains melanocytes, which are usually intermixed among the other components of the tumor [1]. Interestingly, the tumor presented in this case showed the melanocytes clustered at the periphery of the lesion, which is an unusual presentation for melanocytic matricoma. Large clusters of pigmented cells at the periphery and increased mitotic activity simulated melanoma.

In this case, both the clinical and initial histological features of the tumor were suggestive of possible malignant melanoma. Clinically, this was seen by the unusual pigmentation of the lesion. Histologically, this was seen by the presence of large clusters of atypical cells that were heavily pigmented, the presence of many mitoses in the neoplasm, and a high proliferative fraction by Ki67 immunohistochemical stain. The tumor was also discontinuous with the epidermis, which raised concern for metastatic melanoma. It was only on a careful histologic identification of shadow cells and a careful sectioning of the block for a detailed immunohistochemical analysis that the tumor was able to be correctly identified as a melanocytic matricoma. Increased mitotic activity (even up to 14 mitoses/high power field) and high proliferative index (even >70%) are the features described in benign matrical tumors and, therefore, do not indicate malignancy in a melanocytic matricoma, especially in a <2 mm-sized well-circumscribed melanocytic matricoma without necrosis [7,8]. In particular, the pigmented portion of the lesion being positive for melanocytic markers except for PRAME and the non-pigmented basaloid (blue) cell portion of the tumor being positive for LEF1 and beta-catenin were indicative of a melanocytic matricoma and were crucial in ruling out malignant melanoma. Our case illustrates that it is important for pathologists to consider melanocytic matricoma as a possible diagnosis when assessing a lesion for the possibility of malignant melanoma and should be aware of the immunohistochemical profile of this entity.

## Conclusions

Melanocytic matricomas are rare tumors that have yet to be fully understood. That being said, there are certain clinical, histological, and immunohistochemical features that have been elucidated. The case presented in this paper showed a melanocytic matricoma with several unusual features such as atypical-looking melanocytes restricted to the periphery of the lesion in large clusters and the unusually small size of the tumor for a matricoma. Importantly, the initial histological evaluation raised a strong concern for an approximately 2 mm primary malignant melanoma, which is a large melanoma with the potential for metastases or a 2 mm metastatic melanoma to the skin from another site in the body. Careful histologic examination with the detection of a central cluster of shadow cells and immunohistochemical analysis ruled out malignant melanoma and confirmed melanocytic matricoma. As such, it is crucial that pathologists understand the morphological and immunohistochemical features of melanocytic matricomas and keep the possibility of this tumor in the differential when malignant melanoma is suspected.

## References

[1] Ferrier M, Husain A (2022). Melanocytic matricoma: a report of three cases, review of the literature, and suggestion of a new terminology. J Cutan Pathol.

[2] Carlson JA, Healy K, Slominski A, Mihm MC Jr (1999). Melanocytic matricoma: a report of two cases of a new entity. Am J Dermatopathol.

[3] Tumminello K, Hosler GA (2018). CDX2 and LEF-1 expression in pilomatrical tumors and their utility in the diagnosis of pilomatrical carcinoma. J Cutan Pathol.

[4] Perez C, Debbaneh M, Cassarino D (2017). Preference for the term pilomatrical carcinoma with melanocytic hyperplasia. J Cutan Pathol.

[5] Song J, Lu S, Wu Z (2019). An unusual case of melanocytic matricoma in a young pregnant woman. Australas J Dermatol.

[6] Tanboon J, Manonukul J, Pattanaprichakul P (2014). Melanocytic matricoma: two cases of a rare entity in women. J Cutan Pathol.

[7] Sangiorgio V, Moneghini L, Tosi D, Bulfamante GP (2018). A case of melanocytic matricoma with prominent mitotic activity and melanocytic hyperplasia. Int J Dermatol.

[8] Fayyazi A, Soruri A, Radzun HJ, Peters JH, Berger H (1997). Cell renewal, cell differentiation and programmed cell death (apoptosis) in pilomatrixoma. Br J Dermatol.

